# The Effects of Chronic Lifelong Activation of the AHR Pathway by Industrial Chemical Pollutants on Female Human Reproduction

**DOI:** 10.1371/journal.pone.0152181

**Published:** 2016-03-23

**Authors:** Aldo Cavallini, Catia Lippolis, Margherita Vacca, Claudia Nardelli, Alessandra Castegna, Fabio Arnesano, Nicola Carella, Raffaella Depalo

**Affiliations:** 1 Laboratory of Cellular and Molecular Biology, Dept. Clinical Pathology, National Institute for Digestive Diseases, IRCCS “Saverio de Bellis”, via Turi 27, 70013, Castellana Grotte (BA), Italy; 2 Unit of Pathophysiology of Human Reproduction and Gametes Cryopreservation, Dept. of General Surgery, Gynecology, Obstetrics and Anesthesiology, University Hospital of Bari, Consorziale, Policlinico. piazza Giulio Cesare 11, 70124, Bari, Italy; 3 Dept. of Biosciences, Biotechnologies and Biopharmaceutics, University of Bari “A. Moro”, via E. Orabona 4, 70125, Bari, Italy; 4 Dept. of Chemistry, University of Bari “A. Moro”, via E. Orabona 4, 70125, Bari, Italy; University of Oulu, FINLAND

## Abstract

Environmental chemicals, such as heavy metals, affect female reproductive function. A biological sensor of the signals of many toxic chemical compounds seems to be the aryl hydrocarbon receptor (AHR). Previous studies demonstrated the environmental of heavy metals in Taranto city (Italy), an area that has been influenced by anthropogenic factors such as industrial activities and waste treatments since 1986. However, the impact of these elements on female fertility in this geographic area has never been analyzed. Thus, in the present study, we evaluated the AHR pathway, sex steroid receptor pattern and apoptotic process in granulosa cells (GCs) retrieved from 30 women, born and living in Taranto, and 30 women who are living in non-contaminated areas (control group), who were undergoing *in vitro* fertilization (IVF) protocol. In follicular fluids (FFs) of both groups the toxic and essential heavy metals, such as chromiun (Cr), Manganese (Mn), iron (Fe), cobalt (Co), nickel (Ni), copper (Cu), zinc (Zn), cadmium (Cd) and lead (Pb), were also analyzed. Higher levels of Cr, Fe, Zn and Pb were found in the FFs of the women from Taranto as compared to the control group, as were the levels of AHR and AHR-dependent cytochrome P450 1A1 and 1B1; while CYP19A1 expression was decreased. The anti-apoptotic process found in the GCs of women fromTaranto was associated with the highest levels of progesterone receptor membrane component 1 (PGRMC1), a novel progesterone receptor, the expression of which is subjected to AHR activated by its highest affinity ligands (e.g., dioxins) or indirectly by other environmental pollutants, such as heavy metals. In conclusion, decreased production of estradiol and decreased number of retrieved mature oocytes found in women from Taranto could be due to chronic exposure to heavy metals, in particular to Cr and Pb.

## Introduction

Human female reproductive impairment, in terms of reduced fecundity and fertility, has been reported in association with exposures to elevated concentrations of environmental toxic agents that are byproducts of many industrial processes, such as metal production and fuel combustion [1–3].

Unfortunately, the mechanisms of action of these toxic compounds are not always clear because the members of a group of pollutants do not operate through the same mechanism of action and some individual pollutants work through several modes of action.

In female reproduction a biological sensor that responds to the signals of many toxic chemical compounds seems to be the aryl hydrocarbon receptor (AHR) [4].

AHR is a physiological ligand-activated receptor which forms a heterodimer with the aryl hydrocarbon receptor nuclear translocator (ARNT), its cofactor.

After translocation from the cytoplasm to the nucleus, the AHR/ARNT complex binds dioxin-responsive element (DRE), an eight-nucleotide motif located on the promoter of several target genes. Two well-studied members of the AHR-regulated genes, *cytochrome P450 1A1* (*CYP1A1*) and *1B1* (*CYP1B1*), are involved in detoxification of harmful toxicants.

AHR can be activated by toxic chemicals with species- and tissue-specific effects [5]. AHR activated by its ligands, such as dioxins, or other toxic compounds, such as heavy metals, lead to differences in AHR-dependent gene expression profiles [6, 7]. Therefore total action of AHR and AHR battery genes represent a pivotal event in the cell proliferative process and apoptosis cascade [8].

Recently, a group of proteins, termed progesterone receptor membrane component 1 (PGRMC1) and serpine-1 mRNA-binding protein 1 (SERBP1), have been shown to regulate the apoptosis in granulosa cells (GCs) through the binding of progesterone (P4) with PGRMC1 [9].

Since the impact of persistent environmental chemicals on human reproduction is a topic of considerable interest, we have compared, in a case-control study, women born and living in an industrialized and highly polluted area of Italy (Taranto) with women living in non-pollutant areas to evaluate the industrial chemical pollutant effects on granulosa cells (GCs) over a long time. Both groups of women enrolled in this study were undergoing In Vitro Fertilization (IVF) and Embryo Transfer.

After a controlled ovarian stimulation (COS) and oocyte pick up (OPU), the GCs were used to analyze AHR, ARNT, alpha and beta estrogen receptors (ERα and ERβ), progesterone receptor (PR), androgen receptor (AR) levels and the expression of the enzymes related to anabolism (CYP11A1 and CYP19A1) and catabolism (CYP1A1 and CYP1B1) of E2.

The same samples were also used to evaluate the influences of the pollutants on AHR-dependent apoptosis. We analyzed the heavy metals that could represent a longer time of exposure to environmental pollutants [10] in the samples of follicular fluids (FFs).

## Materials and Methods

### Study area and population

Taranto, one of the most highly industrialized cities in Southern Italy, has been defined by the Italian Ministry of Health as pollution-contaminated area revealing several critical situations in terms of mortality [11].

The presence of pollutants was found in the atmosphere, agricultural soil, irrigation water and cattle farm [12–16]. Recently, the dioxin levels in breast milk of lactating mothers living in Taranto were reported [17]. Thus, Taranto infants are exposed to different environmental pollutants, such as heavy metals, during pregnancy via the placenta and postnatally through breastfeeding.

### Study subjects

In our study, 30 women born and living in Taranto, Italy (Taranto Group-TG) and 30 women born and living in rural non-contaminated areas (Non-Taranto Group-NTG) were consecutively enrolled. All participants were undergoing IVF by Intra Cytoplasmic Sperm Injection (ICSI) utilizing autologous fresh oocytes at Reproductive Medicine Unit of University Hospital Bari, Italy.

Inclusion criteria were: (1) age < 39 years; (2) normal menstrual cycle (range of 26–32 days); (3) baseline FSH levels < 12 IU/ml; (4) antral follicle count (AFC) < 10 follicles/ovary; (5) body mass index (BMI) between 18–30 Kg/m^2^; (6) no polycystic ovaries; (7) no oral contraceptive pills taken in the last year and no prior history of low response to ovarian stimulation. Reasons for infertility were male, tubal or unexplained factors. In addition, a short questionnaire was administered to participants of Taranto to obtain demographic and lifestyle information because all those women who had smoked or worked at chemical plants were excluded from the study.

Both groups of women were comparable in terms of age (33.8 ± 2.8 vs. 33.7 ± 2.3 yrs), BMI (23.7 ± 3.6 vs. 21.8 ± 3.03 Kg/m^2^), and Antral Follicle Count (AFC) (14.3 ± 5.5 vs. 14.5 ± 5.04). The baseline detection of Follicle Stimulating Hormone (FSH), E2 and Luteinizing Hormone (LH) did not show any statistically significant difference between the two groups (data not shown).

All subjects could enter the study only once. All participants of this study provide written informed consent, consistent with approval by Institutional Ethics Committee of the University-Hospital, Bari, Italy.

### Ovarian stimulation and IVF procedures

Pituitary desensitization was performed using GnRH antagonists (Cetrotide, Merck Serono, Geneva, Switzerland) administered daily starting when the leading follicle reached a diameter of 14 mm or serum E2 reached 400 pg/mL or GnRH agonists (Decapeptyl 0.1mg/ml, Ipsen SpA, Assago, Milan, Italy) started in the mid-luteal phase of the previous menstrual cycle. Follicular growth was achieved with injectable gonadotropins (rFSH; Follitropin-alpha, Merck Serono, Geneva, Switzerland) and was monitored with transvaginal ultrasound and E2 and P4 serum levels. Recombinant human Chorionic Gonadotrophin (rhCG) (6,500 IU Ovitrelle, Merck Serono) was administered when at least two follicles had a mean diameter of >18 mm to trigger final follicular maturation [18].

Oocyte retrieval was performed 34–36 hours after hCG injection under transvaginal ultrasound guidance. Denuded oocytes were assessed for nuclear status by evaluating the polar body extrusion (MII oocytes), according to Veeck [19]. Mature MII oocytes were inseminated with intracytoplasmic sperm injection (ICSI).

### Collection of GCs and FFs

Samples of follicular fluid (FF) from large follicles (diameter > 16 mm) were collected in separate tube and separately examined to pinpoint differences between immature and mature oocytes. Fluid from each mature follicle was pooled from the same women and GCs were isolated by centrifugation at 1,200 rpm for 5 minutes each at room temperature. Samples that were not clear or were excessively bloody were not assayed for red blood or cellular debris contamination.

The pellets of two FFs obtained from each woman were resuspended in phosphate–buffered saline (PBS) and subdivided in two aliquots. One aliquot was used for RNA extraction and the second aliquot for protein analysis. The two aliquots were then stored at -80°C until analysis.

After centrifugation, the FF supernatants were stored at -80°C until analysis of followed toxic and essential elements: chromium (Cr), iron (Fe), manganese (Mn), cobalt (Co), nickel (Ni), copper (Cu), zinc (Zn), cadmium (Cd) and lead (Pb).

### Nuclear receptor expression

Total RNA was extracted from GCs by TRizol (Life Technologies, Milan, Italy), according to manufacturer’s protocol, and quantified using spectrophotometric assay.

Because of the low concentration of RNA obtained from each aliquot of GCs, a pre-amplification protocol (preAmp) was carried out as previously described [20].

Aliquot of 0.1μg of RNA were retrotranscribed (RT) in 20 μl of reaction by oligo(dT) and iScript cDNA synthesis kit (BioRad, Milan, Italy), according to the manufacturer’s instructions.

Five μl of cDNA were pre-amplified for 5 cycles (PCR condition: 95°C for 2 min, 55°C for 1min, 72°C for 1min) in 50μl of final volume. Five μl of preAmp product was subjected to real-time PCR (qPCR) using the same primers of the preAmp reaction. The specificity of the qPCR product was determined by melting curve analysis. qPCR and melting curve were carried out in an iCycler iQ detection system (BioRad).

Each sample was run in duplicate and, preAmp plus qPCR without retrotranscription was carried out to evaluate the DNA contamination.

Primer sequences, designed by online OligoPerfect Designer (Life Technologies, Milan, Italy), are listed in Table 1.

**Table 1:** Primer sequences of target genes.

Accession number	Gene	Forward (f) and reverse (r) primers	Length amplicon (bps)
NM_001621	AHR	(f) CCTCTGGAAACTCTGGACCTGG	168
		(r) TGGATGGTGGCTGAAGTGGAGT	
NM_001668	ARNT	(f) CTGGCAAACCGCTCCTTATCGT	163
		(r) CCGTTCCCTACCGCCTCCACTCC	
NM_000125	ERα	(f) TTGGCTAACACAGACATCACCTC	237
		(r) CCTGGGCACCTTTCTCCTTTAGT	
NM_001437	ERβ	(f) ACCTTCCTTTTCAGTGTCTCTCT	137
		(r) GGAGGGCGCATATTCCAGAGC	
NM_000926	PR	(f) GGGCCAAACAGGCACCAAGA	293
		(r) TTTTGCCCATTGACTATTACTTTCC	
NM_000044	AR	(f) TTTCCCTTCAGCGGCTCTTTT	134
		(r) CATGTCGGCCACGGAGTTTC	
NM_000499	CYP1A1	(f) CATGTCGGCCACGGAGTTTC	112
		(r) TGGCCCTGGTGGATTCTTCA	
NM_000104	CYP1B1	(f) CGTGGCCACTGATCGGAAAC	103
		(r) CCCAGGCGGATCTGGAAAAC	
NM_000781	CYP11A1	(f) CGAGGACATCAAGGCCAACG	104
		(r) CCTTCAGGTTGCGTGCCATC	
NM_000103	CYP19A1	(f) TGCTCGGGATCTTCCAGACG	117
		(r) CAGGCACGATGCTGGTGATG	
NM_001101	β-actin	(f) GCGGGAAATCGTGCGTGACATTAAGGAGA	220
		(r) CGTCATACTCCTGCTTGCTGATCCACATCTGC	

Each sample was run in duplicate and the results were normalized by β-actin gene expression.

### Western blotting analysis

AHR, ARNT, ERα, ERβ, PR and apoptotic protein levels were evaluated by Western blot as previously described [21]. In brief, after protein extraction by RIPA buffer (Cell Signaling Tehnology, Beverly, MA, USA), 20 μg of proteins were resolved on SDS–PAGE and transferred to polyvinyldifluoride (PVDF) filter (BioRad). The filter was blocked by 5% (w/v) non-fat dry milk for 2 h at room temperature and then probed with primary antibody overnight at 4°C.

The antibodies, purchased from Cell Signaling Technology (Danvers, MA, USA), were used against the following proteins: AHR (catalogue number: #13790), ERα (#8644), ERβ (#5513), PRA/B (#8757), PGRMC1 (#13856), Bax (#5023), Bcl-2 (#4223), Bcl-xl (#2762), Bid and truncated Bid (tBid) (#2002), phospho-survivin (Thr 34) (#8888), survivin (#2802), β-actin (3700), cleaved caspase-3 (#9664), -7 (#9491), -9 (#9496) and full-length caspase-3 (#9665), -7 (#9494) and -9 (#9508).

MCF7 and MDA-MB-231 human breast cancer cell lines (The European Collection of Authenticated Cell Cultures (ECACC), Salisbury, UK) were used as respectively positive and negative controls for steroid receptors [22].

A human hepatocarcinoma cell line (HepG2; American Type Culture Collection (ATCC), Rockville, MD, USA) and human placental tissue samples, obtained from Dept. of Gynecology and Obstetrician, University of Bari, Italy, were used as positive controls for apoptotic proteins and both AHR and ARNT, respectively [21].

The immunoreactive signals were analyzed and quantized by ChemiDoc XRS Gel Imaging System (BioRad).

Each sample was run in triplicate. AHR, ERα, ERβ, PRA/B, PGRMC1, Bax, and Bcl-2 levels were normalized by β-actin ones. Cleaved proteins were normalized by their full-length proteins, while phospho-survivin was normalized by total survivin proteins.

### Measurements of heavy metals

Quantitative elemental analysis was conducted via inductively-coupled plasma mass spectrometry (ICP-MS) according to Calleja et al. [23]. In brief, the follicular fluid samples were digested at 80°C for 4h with 1 ml of ultrapure solution of H_2_O_2_/HNO_3_ (1:7 v/v) and a final cooling stage of 15 min. The digestion procedure is safe and simple with minimal risk of contamination of the samples during handling and it provides good recoveries of trace elements in FFs.The concentration of selected toxic and essential elements was determined using a Varian 820 ICP mass spectrometer (Varian Inc., Palo Alto, CA, USA).

All samples were analyzed three times. For calibration multielemental standard solutions were prepared from the stock standard solutions (10 mg/L for Mn, Fe, Co, Cu, Zn, Cd; 50 mg/L for Cr and Ni; 100 mg/L for Pb) purchased from Fluka Analytical (Sigma-Aldrich, Milan, Italy). Detection limits were calculated as the concentrations of an element that gave a signal equal to three times the standard deviation of a series of ten successive measurements of the blank solution at the element peak.

All reagents used were of analytical grade.

Since dilution is often required in order to eliminate or reduce non-spectroscopic interferences (matrix effects), the analytical data were normalized to an equal volume of follicular fluid.

### Statistical analysis

Differences between two groups were evaluated by Mann-Whitney test and were considered significant for *P* value < 0.05.

All statistical analyses were performed by Graph Pad Prism 5.0 software (San Diego, CA, USA).

## Results

### Participants

At the end of ovarian stimulation (hCG day administration) the serum estradiol (E2) levels were 2,333.6 ± 1,810.8 vs. 3,531.8 ± 1,861.3 pg/ml (mean ± SD, *P* = 0.03) and P4 levels were 1.41 ± 0.83 vs. 1.48 ± 0.66 nmol/l (mean ± SD, *P* = 0.4) in TG vs. NTG, respectively. A significantly lower number of mature oocytes (MII) was retrieved in women of Taranto compared to control group (6.7 ± 3.8 vs. 9.5 ± 3.5, *P* = 0.03).

## Nuclear receptor and aromatase mRNA expression

**Fig 1:**
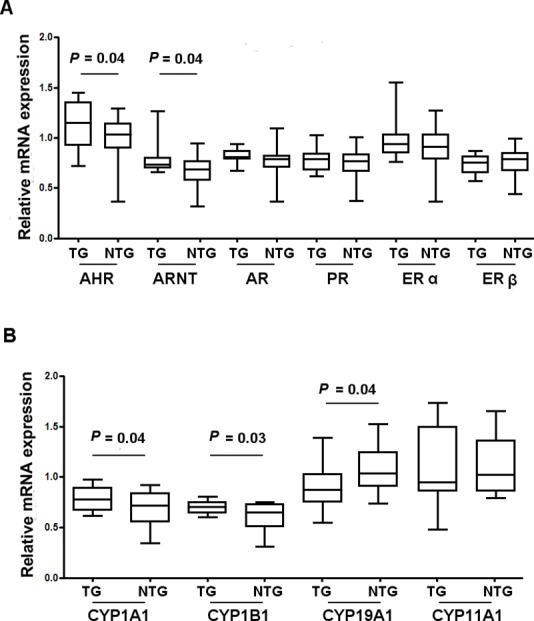
Nuclear receptor and cytochrome P450 enzyme levels in granulosa cells. **(A)** Relative mRNA expression of aryl hydrocarbon receptor (AHR) and aryl hydrocarbon receptor nuclear translocator (ARNT) were higher in granulosa cells of women of Taranto (TG) as compared to ones of non-Taranto group (NTG). The androgen (AR), progesterone (PR) and estrogen (ERs) receptors had the same levels in both groups. (**B)** Relative mRNA expression of two aromatase (CYP1A1 and CYP1B1), targets of AHR, were higher in granulosa cells of TG versus NTG, while CYP19A1 was lower in TG. Data are presented as box and whisker plots.

qRT-PCR analysis in GCs showed a significant increase of AHR (1.14 ± 0.22 vs. 0.98 ± 0.24, TG vs NTG, *P* = 0.04) and for ARNT (0.78 ± 0.14 vs. 0.66 ± 0.17, TG vs. NTG, *P* = 0.04) mRNA expression, while no differences were observed for steroid receptor (ERα, ERβ, AR and PR) expression between two groups (Fig 1A).

The cytochrome P450 mRNA levels in GCs differed between the wo groups. CYP1A1(0.79 ± 0.11 vs. 0.69 ± 0.17, *P* = 0.04) and CYP1B1 (0.70 ± 0.06 vs. 0.61 ± 0.14, *P* = 0.03) expression increased in GCs of TG vs. NTG. By contrast, CYP19A1 mRNA levels decreased in TG than in NTG (0.80 ± 0.20 vs. 1.03 ± 0.222, *P* = 0.04). When CYP11A1 mRNA levels were assessed, both groups showed the same levels (Fig 1B).

### Protein analysis

**Fig 2:**
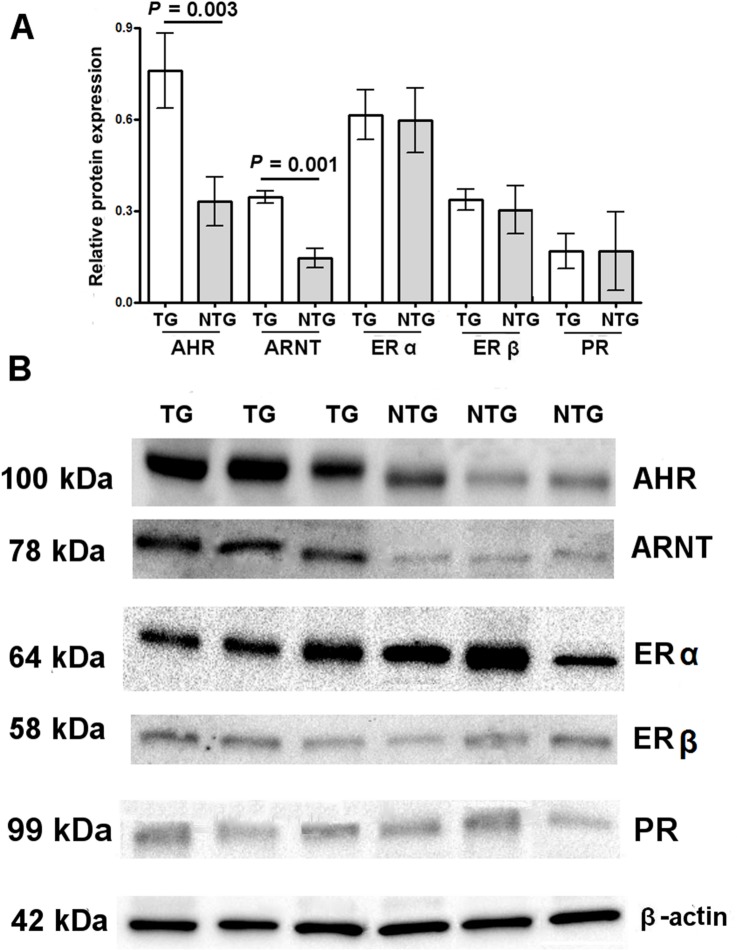
Western blot analysis of the nuclear receptors in granulosa cells. **(A)** Western blot analysis confirmed the same distribution of the nuclear receptor proteins found by real-time PCR (Fig 1A). Each sample was run in triplicate. Data are presented as mean ± SD. **(B)** Representative Western blots showing nuclear receptor levels in both groups of women.

The Western blot analysis confirmed at the protein level the findings concerning AHR, ARNT, ERα, ERβ and PR at the mRNA expression (Fig 2A and 2B).

**Fig 3:**
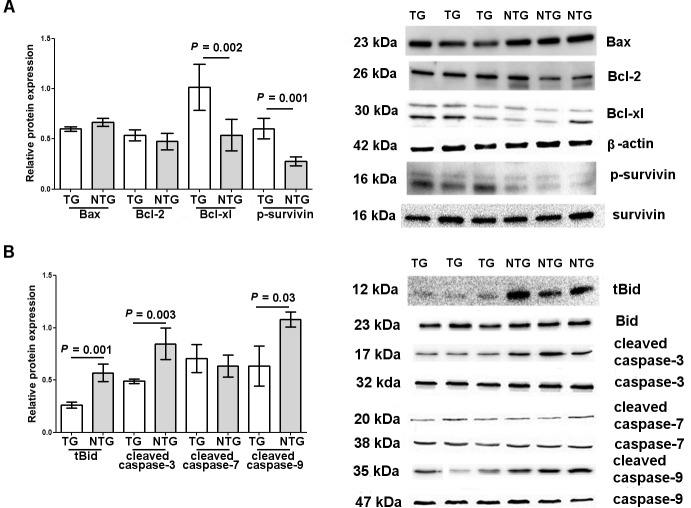
Relative apoptotic protein content in granulosa cells after ovarian hyperstimulation. Distribution of (**A**) anti-apoptotic (Bcl-2, Bcl-xl, phospho-survivin) and (**B**) pro-apoptotic (Bax, truncated Bid (tBID) and caspases) proteins in granulosa cells of both groups of women. Bcl-2, Bcl-xl and Bax were normalized by β-actin levels. Phospho-survivin was normalized by total survivin expression. Cleaved caspases and truncated Bid (tBid) were normalized by their full-length proteins. Each sample was run in triplicate. Data are presented as mean ± SD. On the right, representative Western blot analysis showing pro- and anti-apoptotic proteins.

For apoptosis, tBid, cleaved caspase-3 and -9, three pro-apoptotic proteins, were statistically decreased in TG vs. NTG. In contrast, Bcl-xl and phospho-survivin, two anti-apoptotic proteins, were statistically increased in TG than in NTG. (Fig 3A and 3B).

**Fig 4:**
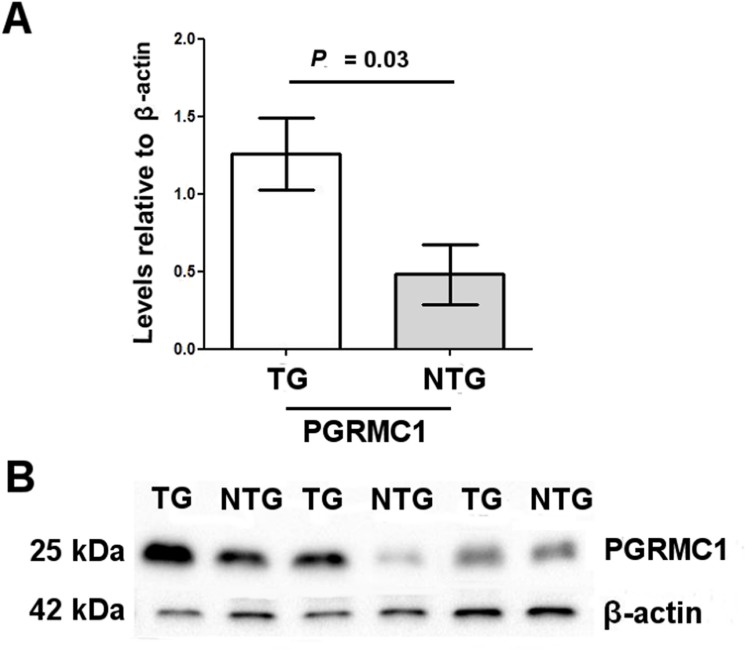
Progesterone receptor membrane component 1 (PGRMC1) proteins in granulosa cells of two groups of women. (**A**) Higher levels of PGRMC1 proteins in TG versus NTG. Each sample was run in triplicate. PGRMC1 levels were normalized by β-actin expression. Data are presented as mean ± SD. **(B)** Representative Western blot analysis showing PGRMC1 expression in both groups of women.

PGRMC1 levels showed a significant increase in TG than in NTG (1.26 ± 0.52 vs. 0.48 ± 0.38, *P* = 0.04,respectively) (Fig 4).

### Heavy metal concentrations in FFs

The heavy metal amount in both groups of women was: Cu > Fe ≥ Zn >> Cr ≥ Ni > Pb ≥ Mn ≥ Co, whereas the cadmium amount was present only in trace in follicular fluids (data not shown).

The levels (median ± SD) of FF elements, expressed as ng/ml, were statistically higher in TG vs. NTG: Fe (621,60 ± 380.50 vs. 304.70 ± 153.10; *P* = 0.042), Zn (578.50 ± 341.60 vs. 284.60 ± 102.50; *P* = 0.029), Cr (7.90 ±3.11 vs. 5.52 ± 0.52; *P* = 0.013) and Pb (2.00 ± 2.01 vs. 0.68 ± 0.22 *P* = 0.003).

No difference (means ± SD) between the two groups of women were found for Cu (993.10 ± 259.90 vs. 970.90 ± 312.40; *P* = 0.95), Ni (6.67 ± 3.92 vs. 5.25 ± 1.12; *P* = 0.90), Mn (1.58 ± 0.67 vs. 1.54 ± 1.3; *P* = 0.34) and Co (1.24 ± 1.51 vs. 0.65 ± 0.11; *P* = 0.66).

## Discussion

Various exogenous environmental pollutants can influence female reproductive health, pre- and postnatal development, by mechanisms not always well known and many studies have shown a significant relationship between heavy metal contamination in the follicular fluid and negative IVF outcomes [24–26].

The major sources of heavy metal pollution are industrial processes, such as smelting of non-ferrous ores, waste incineration and the disposal of sewage sludge onto land, that release toxic compounds into the environment resulting in enhanced levels of many heavy metals.

Specific studies of assessment of heavy metals in soil, air and sea (Taranto Gulf) of Taranto city have been carried out [14, 15, 27]. Therefore, Taranto has been included in the list of polluted sites of national interest in order to implement environmental remediation.

A large mortality analysis was conducted within this hot spot area, evidencing a significant excess of mortality for many and different tumors (11). However, the impact of the chronic exposure of the toxic compounds, in particular heavy metal exposure, on female fertility has never been described.

Our hypothesis was that the highest metal ion concentrations within follicles of women from Taranto represented a long-term exposure to environmental pollutants (chronic exposure) and that these elements could affect the follicular development in women undergoing IVF.

Similar to findings by Silberstein et al. [10], our data showed that the most abundant elements found in FFs in both contaminated and non-contaminated women were copper (Cu), iron (Fe) and zinc (Zn), followed by chromium (Cr), nickel (Ni), lead (Pb), manganese (Mn) and cobalt (Co). In particular, Fe, Zn, Cr and Pb levels were significantly higher in women from Taranto as compared to controls.

We were unable to elaborate a comparison between our data and those of previous studies because of a lot of differences existing among the countries involved in the study, the types of heavy metals measured or levels and time of human exposure or simply because of the different analytical procedures used.

We have focused our attention on AHR pathway. Several studies have addressed heavy metals as modifiers of CYP450 system, but an involvement of these elements, especially Pb, on the AHR expression and activity is still today debated [5, 28]

Recently, Darwish et al. reported that Pb slightly induced AHR expression, as well as AHR-DRE activity, in HepG2 cells under the low Pb concentrations. On the contrary, higher Pb concentrations significantly reduced AHR expression. This suggests that the modulatory effects of Pb on AHR-mediated genes are possibly due to Pb-AHR crosstalk [29].

The women from Taranto were subjected to chronic exposure of pollutants and this type of exposure, in opposite to acute exposure, is characterized by a continuous contamination of low levels of toxic substances, over an extended period. Therefore, a chronic exposure to low lead levels might exert a potential toxicity on GCs through an AHR/CYP1A1-mediated mechanism.

Our data showed that the AHR and ARNT mRNAs were up-regulated in GCs of the women from Taranto and this phenomenon was associated to an increase in CYP1A1 and CYP1B1 expression, two enzymes involved in E2 catabolism, and a decrease of CYP19A1, a key enzyme involved in E2 biosynthesis.

Since E2 is critical for normal follicle development, the reduction of aromatase function led to the inability to convert androgens to estrogens resulting in a decrease in estrogen production in women from Taranto at the end of the ovarian stimulation. The serum E2 depletion could explain the negative effect on the folliculogenesis leading to a reduced number of mature oocytes retrieved.

Several studies support, at least in part, our data. Pillai et al. [28] and Nampoothiri et al. [29] report that treatment of both lead and cadmium alone and in combination in a animal model decrease serum levels of estradiol and progesterone and this may be attributed to the inhibition of ovarian steroidogenic enzyme activity. Moreover, the decreased levels of ovarian steroids in metal-treated animals were also correlated with decreased numbers of follicles and the granulose cell population in the developing follicles.

Another study clearly demonstrated that lead, accumulated in cultured human granulosa cells, exerted its function by down-regulation of P450 aromatase enzymes and ERβ gene transcription [30]. However, we observed no differences between two groups of women in steroid receptor levels suggesting that the steroid receptors are not influenced by lead pollution.

The consequences of Pb exposure on female fertility described by both in vitro and/or animal model were also supported by epidemiological studies in women undergoing to IVF [26, 31].

Lead exposure in early life may be a risk factor for fetuses and the adverse effects occur also with low-level lead exposure [32]. If we consider the average age of women from Taranto and the beginning of the process of pollution in this geographic area, we can think that heavy metal exposure in these women occurred since prenatal period.

More recently, an in vitro study reported that chromium exposure, as well as lead exposure, actives the DNA-binding capacity of the AHR on the DRE with a consequent increase of Cyp1A1 mRNA levels by both transcriptional and post-transcriptional mechanisms [33]. In addition, chromium element acts on cell proliferation and apoptotic process with relative DNA fragmentation in primary culture of rat granulosa cells [34, 35].

The results of the present study show an increase of anti-apoptotic proteins associated with an increase of PGRMC1 proteins in women from Taranto as compared to controls. PGRMC1 is known to promote the P4’s anti-apoptotic action in human granulosa cells [36] and, among other functions, it seems to have the ability to interact with the mitotic spindle apparatus in GCs [37].

Both anti-apoptotic and anti-mitotic processes, associated with a decrease of E2 production, could explain the lower number of mature oocytes (MII) in women from Taranto.

The PGRMC1 role in promoting ovarian cell survival has also a clinical relevance because depletion, methylation or point mutations of its promoter have been described in women with Premature Ovarian Failure (POF) [38].

Gonadotropin-induced follicle development in women undergoing IVF is attenuated by the presence of high PGRMC1 levels, despite of a sufficient numbers of follicles [9]. This re-enforce the concept of PGRMC1 inhibitory effect on the mitotic process of the GCs.

Although all metals have inherent toxic properties, some elements, as Fe and Zn, are important in human metabolism.

Zinc increases in the oocyte during maturation and it is important for progression and completion of meiosis I. However, the metabolic mechanism of the zinc homeostasis in oocytes is still unknown.

During ovarian stimulation the oxidative stress with a relative increase in reactive oxygen species (ROS) occurs as consequence of the folliculogenesis [39].

Most ROS are formed physiologically as consequence of the activity of the mitochondrial respiratory chain, but can also be formed by environmental pollutant exposure and among them certain metallic cations, such as copper and iron, may contribute significantly to ROS generation [40].

Under normal conditions, cells generate ROS from which they protect themselves through the action of several antioxidant enzymes (e.g., superoxide dismutase or glutathione reductase), but when the normal oxidative status is perturbed, cell damages may occur [41].

Therefore, the increased levels of zinc, as anti-ROS factor, and of iron, as ROS generator, in FFs of women from Taranto may be two consequences of the chronic exposure to heavy metals.

However, studies into the effects of metallic elements on the reproductive system have generally involved a single metal, but the human exposure effects are much more complex because a single individual is simultaneously exposed to several metals and organic compounds that may have additive, synergistic or antagonist effects.

In conclusion, our data suggest that heavy metals, especially chromium and lead, may induce a negative effects on follicular maturation in women undergoing IVF similar to that which is observed with classical AHR ligands, such as dioxins.

Additional in vitro studies should be conducted on the granulosa cells to obtain information about the relationship between AHR activation and heavy metals.
